# Case Report: Pancreatic metastasis of renal cell carcinoma 16 years after nephrectomy

**DOI:** 10.3389/fonc.2023.1091635

**Published:** 2023-02-09

**Authors:** Yidan Lou, Kaibo Guo, Song Zheng

**Affiliations:** ^1^Department of Oncology, Affilited Hangzhou First People’s Hospital, Zhejiang University School of Medicine, Hangzhou, China; ^2^Zhejiang University School of Medicine, Hangzhou, China; ^3^Department of Oncology, The Fourth School of Clinical Medicine, Zhejiang Chinese Medical University, Hangzhou, China; ^4^Department of Oncology, Affiliated Hangzhou Cancer Hospital, Zhejiang University School of Medicine, Hangzhou, China; ^5^Key Laboratory of Clinical Cancer Pharmacology and Toxicology Research of Zhejiang Province, Affiliated Hangzhou First People’s Hospital, Zhejiang University School of Medicine, Hangzhou, China

**Keywords:** renal cell carcinoma (RCC), late recurrence, genetics, VHL, Pten, KDM5C, isolated pancreatic metastasis

## Abstract

**Background:**

Renal cell carcinoma (RCC) is the most common renal malignancy, and may metastasize to different sites in the body *via* hematogenous and lymphomatous routes. The pancreas is a rare metastatic site of metastatic RCC (mRCC) and isolated pancreatic metastasis of RCC (isPMRCC) is even rarer.

**Results:**

The present report describes a case of isPMRCC that recurred 16 years after surgery. The patient responded well to the treatment with pancreaticoduodenectomy and systemic therapy, and no recurrence was recorded after 2 years.

**Conclusions:**

isPMRCC is a distinct subgroup of RCC with unique clinical characteristics that may be explained by its underlying molecular mechanisms. Surgery and systemic therapy confer survival benefits to patients with isPMRCCs, although the recurrence problem has to be paid attention to.

## Introduction

Tumors of the renal and perirenal tissues account for 2% to 3% of all malignancies, and among these, renal cell carcinoma (RCC) accounts for 85% of all renal parenchymal tumors (1). The metastasis of RCC is sporadic, with the metastatic sites including the lung, bone, adrenal gland, liver, kidney, brain, etc. (2) RCC may also metastasize to rare sites, such as the duodenum, and the intestine (3, 4). However, pancreatic metastasis (PM) of RCC is quite rare (5). PM may appear in two clinical manifestations:(1) isolated pancreatic metastasis and (2) in the context of multi-organ metastatic disease. Cases of isolated pancreatic metastasis are extremely rare, accounting for approximately 1% to 3% of diagnoses (6).

The present report describes the case of a patient who developed the isolated pancreatic metastasis of renal cell carcinoma (isPMRCC) at the head of the pancreas 16 years after the curative resection.

## Case presentation

**Figure 1:**
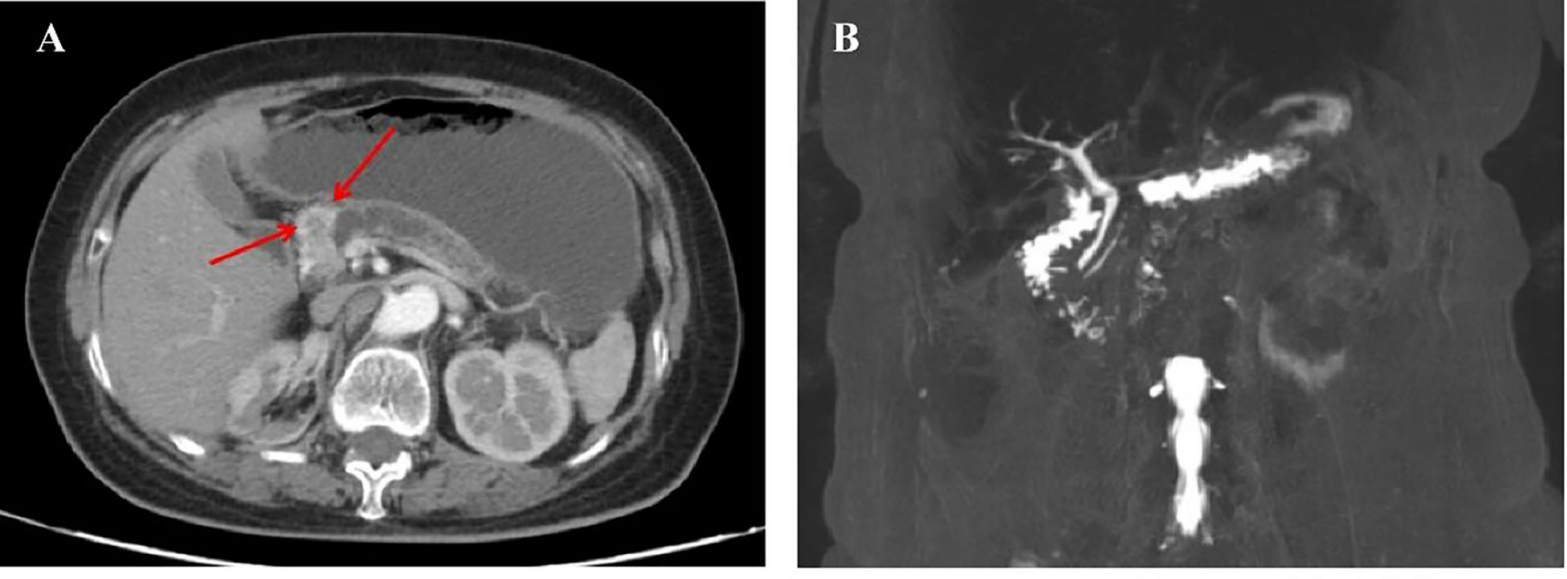
**(A)** An Abdominal computed tomographic scan and contrast-enhanced scan taken on March 17, 2020, showing a mass in the head of the pancreas, with atrophy of the body and tail of the pancreas, and the pancreatic duct obviously dilated. **(B)** Magnetic Resonance Cholangiopancreatography imaging examination taken on March 19, 2020, showing a space-occupying lesion in the head of the pancreas, with obvious dilation of the pancreatic duct at the tail of the pancreas.

A 73-year-old woman was admitted to our hospital with abnormal imaging results and no symptoms. Contrast-enhanced CT scan (**Figure 1**) revealed a mass in the head of the pancreas, with atrophy of the body and the tail of the pancreas, and the pancreatic duct was dilated, which suggested pancreatic cancer. Magnetic Resonance Cholangiopancreatography (**Figure 1**) revealed a space-occupying lesion in the head of the pancreas. The physical examination of the abdomen was negative. The past medical history of the patient revealed that she had undergone a right partial nephrectomy for renal cell carcinoma 16 years earlier. At the time of the surgery, the clinical stage of renal cell carcinoma was stage I (T1aN0M0), according to the TNM classification. Pathology revealed a Fuhrman grade 3 clear cell RCC (ccRCC). The patient did not receive adjuvant chemotherapy or targeted therapy later. Rather, they were followed up for abdominal computed tomography (CT) and ultrasonography (US), which revealed no evidence of recurrent disease prior to the present admission at this hospital. A further metastatic workup, including Positron Emission Tomography/Computed Tomography (PET/CT), did not disclose any evidence of extra pancreatic lesions.

**Figure 2:**
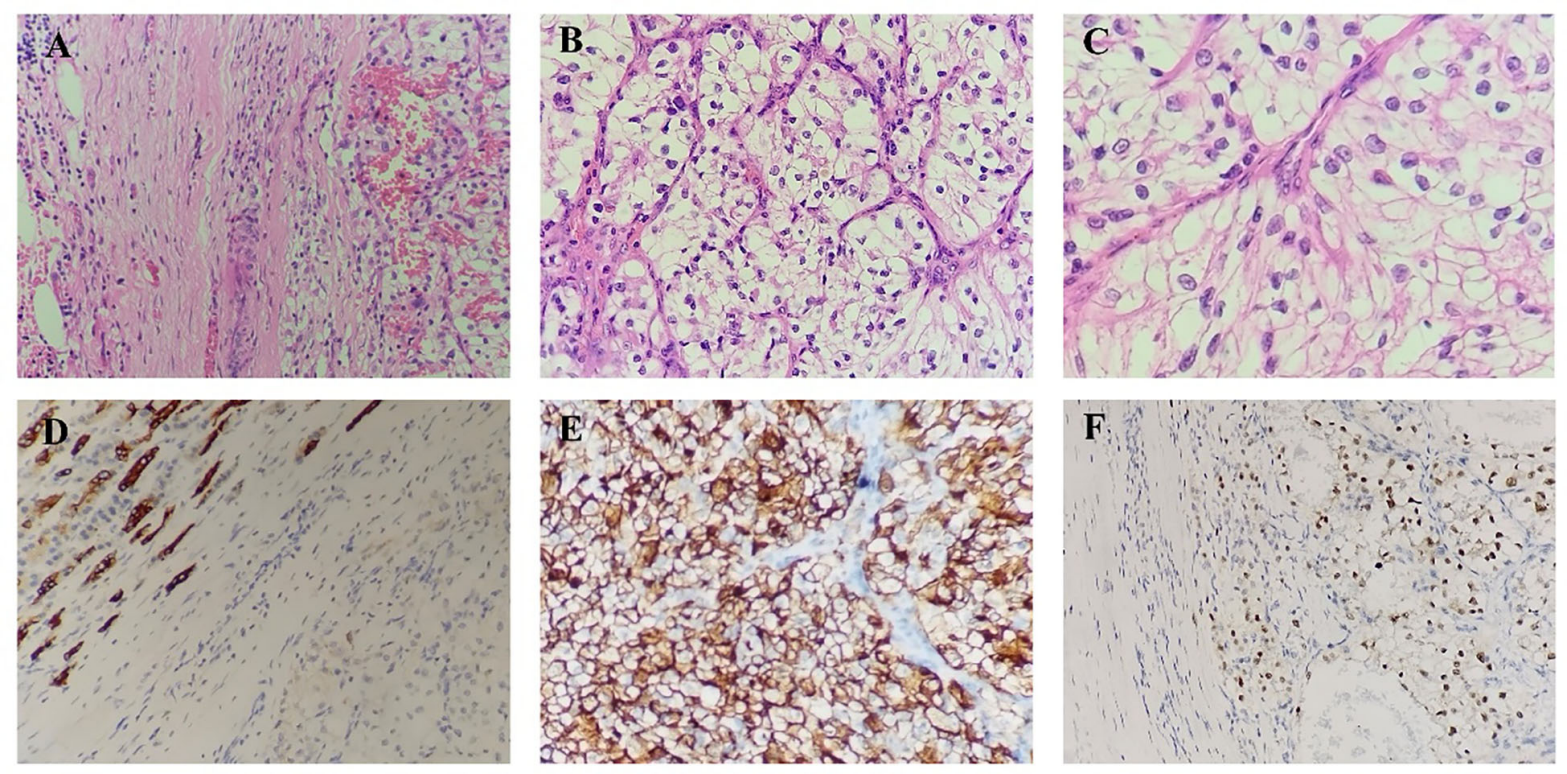
**(A–C)**. Microscopic picture with hematoxylin and eosin staining showing the renal cell carcinoma within the pancreatic tissue. **(D)**. Immunohistochemistry staining of the resected lesion from the pancreas, showing positive CK. **(E)**. Immunohistochemistry staining of the resected lesion from the pancreas, showing positive CD10. **(F)**. Immunohistochemistry staining of the resected lesion from the pancreas, showing positive PAX-8.

**Figure 3:**
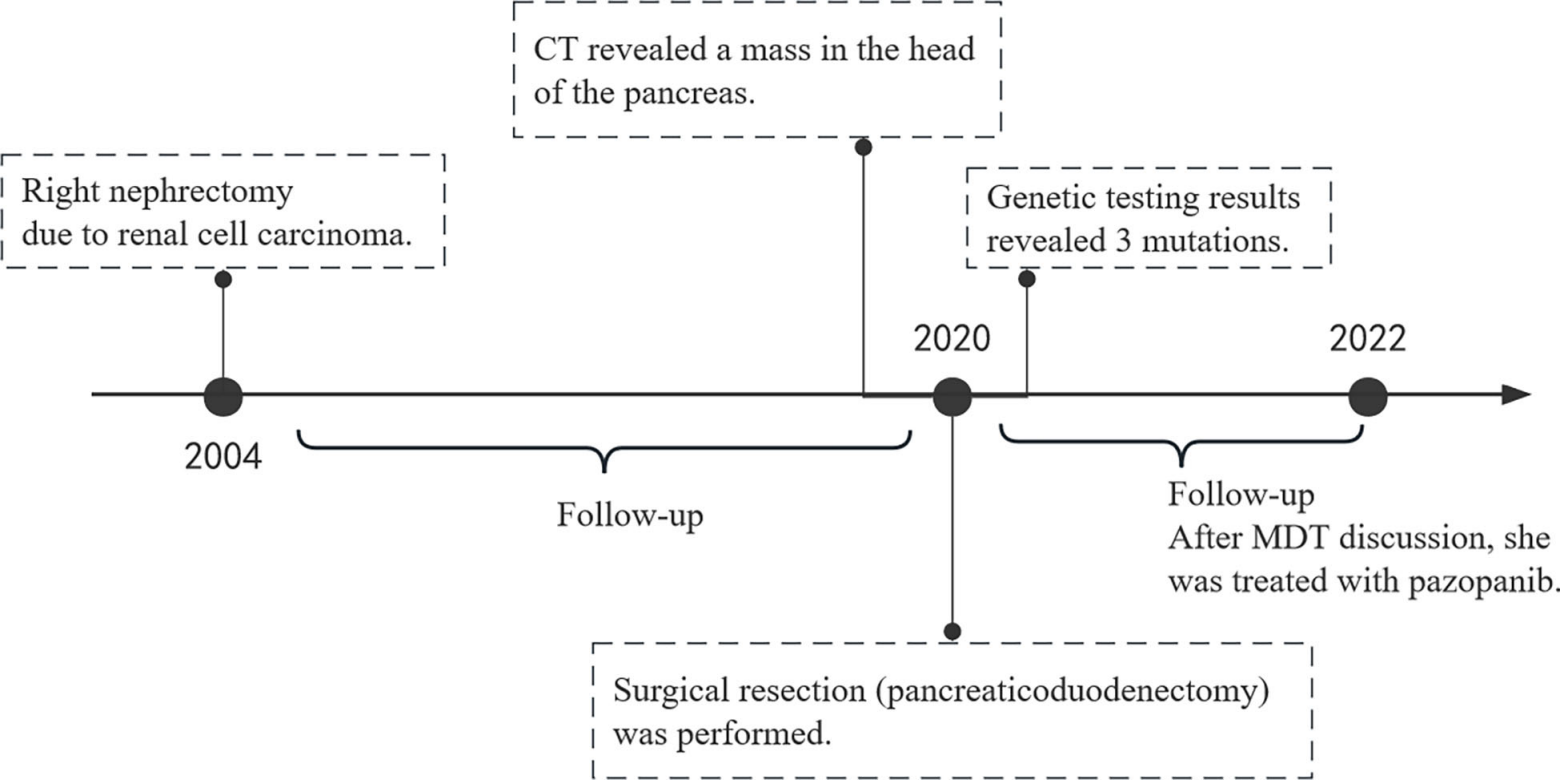
timeline of our patient.

The possibility of a neuroendocrine tumor was considered, and metastasis from renal cancer could not be excluded. There were indications for surgery according to the NCCN guidelines, and the specific surgical approach was determined based on the intraoperative mass location and intraoperative rapid frozen pathology. Pancreaticoduodenectomy was performed. The surgical specimen demonstrated a 3*2.5*2cm tumor in the head of the pancreas, which was consistent with metastatic renal cell carcinoma and morphology, history, and immunohistochemistry analyses. No tumor involvement was detected in the common bile duct incision margin, pancreatic head stump margin, upper gastric incision margin, and inferior duodenal incision margin. No cancer metastasis was observed in the surrounding lymph nodes, although there was a tumor thrombus in small intratumoral vessels. The microscopic image examination after hematoxylin and eosin staining (**Figures 2A–C**) revealed renal cell carcinoma within the pancreatic tissue. Histopathological examination (**Figures 2D–F**) revealed paired box gene 8-positive (PAX-8+), cluster of differentiation 10-positive (CD10+) and creatine kinase-positive (CK+) cells. According to the patient’s medical history, histopathological examination, clinical images, and pathological findings, a final diagnosis of isolated pancreatic metastasis of renal clear cell carcinoma was established. The outcomes of next-generation sequencing (NGS) verified three gene mutations (**Table 1**) in von Hippel-Lindau (VHL), phosphatase and tensin homolog (PTEN), and lysine-specific histone demethylase 5C (KDM5C), a low tumor mutation burden (TMB) of 6.3 Muts/Mb, and a microsatellite stable (MSS) status. A multidisciplinary team (MDT) discussed the case, and the patient was then treated with pazopanib-targeted therapy after surgery to prevent tumor recurrence. The patient was followed in the outpatient clinic for over 2 years, and no recurrence was reported. The timeline of the case is presented in **Figure 3**.

**Table 1:** The genetic testing results.

Gene	Variants			Frequency
VHL	N90Ffs*70	exon6	Indel	12%
PTEN	P231Rfs*22	exon7	Indel	8%
KDM5C	Y521*	exon11	Indel	12%

## Discussion

The common sites for the metastasis of RCC are the lungs, liver, lymph nodes, and brain (2). The pancreas is one of the rare sites of RCC metastasis, accounting for < 2% of patients with advanced disease (7). Isolated metastasis from RCC is rare, with an incidence of between approximately 1% and 4% for cases with pancreatic origins (7). In terminal systemic disease tumors, pancreatic metastasis rarely occurs, in approximately 2% of surgical cases, with a corresponding poor prognosis (8, 9). However, among patients with multi-organ site mRCC, concomitant PM presents a better prognosis compared to the cases without PM (median OS: 42 months vs. 23 months) (10). Furthermore, isPMRCC is characterized by an unusually favorable prognosis after surgical therapy.

This type of tumor presents low tumor aggressiveness, which results in a long interval between the initial diagnosis and the metastatic disease. The median interval is 7.1-10.0 years (11), and PM from RCC may occur over 36 years after the primary diagnosis. In the case presented here, metastasis to the head of the pancreas was detected during imaging surveillance 16 years after the resection of the primary tumor. This kind of metachronous metastasis is rare. Certain researchers suggest that such patients harbored ccRCC micro metastasis in the tumor microenvironment (TME), which then gradually established at the PM site and released cytokines that promoted angiogenesis and induced immune tolerance (12, 13). Moreover, a chronic state of low-grade inflammation due to long-term endogenous or exogenous factors could induce tumor progression (14). Notably, most of these patients are asymptomatic, and the diagnosis is usually incidentally or established during radiological surveillance (15, 16). Owing to the long interval and atypical clinical symptoms, an early diagnosis becomes challenging. Therefore, patients with a history of RCC require a meticulous long-term follow-up to ensure that recurrence is not missed.

As a distinct case of RCC, isPMRCC has a clinical hallmark of confinement to the pancreas while the other organs are left unaffected for a long duration. This kind of organotropism may be explained based on seed and soil theory, according to which the metastatic process is determined by the interaction between the tumor cells (seed) and the host organism (soil) (17, 18). Research on microRNA has identified that miRNAs regulate the metastatic potential of tumor cells and explain the seed and soil concept biochemically (17, 19). Their interactions are related to genetic changes associated with the activation of proto-oncogenes or inactivation of suppressor genes (20), such as PTEN, and may involve interactions with the tumor microenvironment, the immune system, and altered epigenetic status (21).

**Figure 4:**
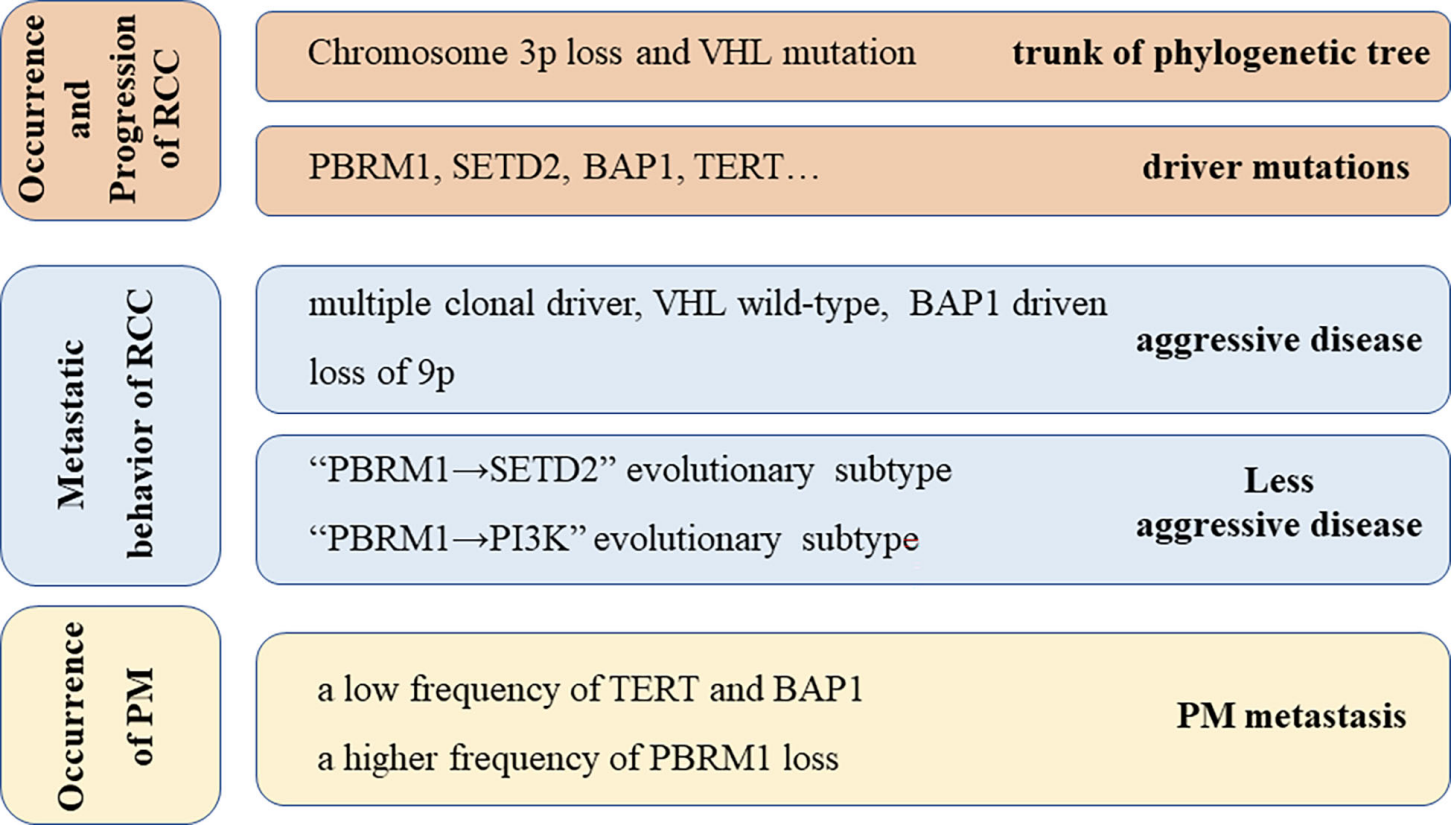
Underlying Genetic alterations possibly responsible for the behavior of RCC and PM metastasis, including genetic alterations responsible for the occurrence and progression of RCC (23), metastatic behavior of RCC (24), and occurrence of PM (25).

Certain possible biochemical mechanisms are reported for the characteristics of RCC. It is reported that the genome of ccRCC is distinctive. In ccRCC, the presence of some mutations is common, as reported in a retrospective review (N=105) (22). The most frequent mutations were detected in VHL (83%), PBRM1(51%), SETD2(35%), BAP1(24%), KDM5C (16%), and TERT (14%). Notably, chromosome 3p deletions and VHL mutation are always at the trunk of the phylogenetic tree, which suggests that these two kinds of mutations are key early events in the development of RCC. Turajlic et al. were the first highlight the genetic changes relevant to the metastatic behaviors of renal cell cancer and also discuss the evolutionary classification of different metastatic potentials (**Figure 4**) (24). Concerning pancreatic metastasis, gene targeting experiments in mouse kidneys revealed that the inactivation of VHL and PBRM1 resulted in ccRCC with a prominent vascular network and compact nests, which indicates that these alterations probably drive the characteristic histology of PM. However, the inactivation of VHL and BAP1 resulted in further aggressive, inflammatory, and less-vascularized tumors. Singla et al. (25) reported that certain specific genetic changes in the PM cell clones are associated with less aggressive disease, including a low frequency of copy number alterations, a low frequency of BAP1 mutations, and a high frequency of PBRM1 loss. In the case presented here, no specific BAP1 gene mutation was detected, which suggested gradual tumor growth (25).

In the present case, genetic testing was performed, and the resulting profiling data revealed gene mutations in VHL, PTEN, and KDM5C. VHL mutation is a dominant feature in ccRCC. PTEN is recognized as a dormant tumor suppressor, and a loss of PTEN expression may result in the initiation of tumorigenesis (26). Mutations in both VHL and PTEN are reported to be significantly associated with a poor prognosis (27, 28). In contrast, mutations in KDM5C play a functional role in the epigenetic phenomena (29). KDM5C is a chromatin modifier gene that encodes demethylases that target trimethylated lysine 4 on histone H3. Chromosomal dysfunction may lead to disease development. A study reported that tumors with a mutation in KDM5C are statistically associated with a higher stage (30). In another study, tumors with KDM5C mutation presented heterochromatin and genomic rearrangement, which resulted in a poor prognosis (29). In addition, studies have demonstrated that a mutation in this gene is associated with a higher risk of cancer recurrence and death among patients with small renal masses (30, 31). One main reason for this is that this mutation is related to the distribution of TAMs, which reportedly support tumor cell growth and metastasis through a series of pathways (32). Therefore, a metastatic event could be reasonably suspected in an RCC patient with a KDM5C mutation. Therefore, it was postulated that the occurrence, proliferation, and metastatic behavior of ccRCC could be explained based on the related genome alteration.

Pancreatoduodenectomy was performed in the present case, and no recurrence was detected during follow-up for 2 years after surgery. Pancreatectomy for metastasis is not common, although RCC is the most common primary source (33). A survival benefit is associated with this subset of patients when they undergo surgery (complete oncological resection is associated with a 5-year survival rate of 75%) (10), particularly if RCC is the primary source (34). However, it has been reported that after the treatment of isPMRCC, 41.5% of patients present recurrence (125 patients among 301 patients relapsed after a 30-month interval) (10). The longest reported interval for recurrence was 10 years. Therefore, long-term monitoring is recommended. Since mRCC has a poor outcome overall, targeted therapy (pazopanib) has also been used after surgical treatment to improve the patient’s survival, and the patient exhibited a good response. Pazopanib is a novel TKI that inhibits the vascular endothelial growth factor receptor, platelet-derived growth factor receptor, c-Kit, and other targets. Currently, pazopanib has been approved in the EU, the USA, and other nations for the treatment of advanced RCC (35). In addition, several prospective clinical trials and retrospective studies support the use of pazopanib as a standard or alternative first-line treatment for advanced or metastatic RCC (35–37).

## Conclusions

isPMRCC is a distinct subgroup of RCC with unique clinical characteristics that may be explained based on underlying molecular mechanisms. Surgery and systemic therapy present survival benefit in patients with isPMRCCs, although the recurrence problem should be paid attention to.

## Data availability statement

The original contributions presented in the study are included in the article/supplementary material. Further inquiries can be directed to the corresponding author.

## Ethics statement

Written informed consent was obtained from the individual(s) for the publication of any potentially identifiable images or data included in this article.

## Author contributions

YL, KG collected the data; YL, KG, and SZ wrote the manuscript. All authors read, provided feedback, and approved the final version.
